# Changes in Serial D-Dimer Levels Predict the Prognoses of Trousseau's Syndrome Patients

**DOI:** 10.3389/fneur.2018.00528

**Published:** 2018-07-03

**Authors:** Shinji Ito, Koichi Kikuchi, Akihiro Ueda, Ryunosuke Nagao, Toshiki Maeda, Kenichiro Murate, Sayuri Shima, Yasuaki Mizutani, Yoshiki Niimi, Tatsuro Mutoh

**Affiliations:** Department of Neurology, Fujita Health University School of Medicine, Aichi, Japan

**Keywords:** Trousseau's syndrome, D-dimer, acute multiple embolic infarction, atrial fibrillation, cancer, prognosis

## Abstract

**Background:** The development of acute multiple embolic infarctions (AMEI) resulting from cancer is known as Trousseau's syndrome (TS). At present, however, there is no good marker for predicting the prognosis of TS patients. In the present study, we evaluated the use of serial D-dimer levels as a prognostic marker for TS.

**Methods:** This retrospective cohort study included 1,409 consecutive acute ischemic stroke patients. We selected a group of patients with TS showing AMEI (*n* = 38; TS group) and a group of patients with atrial fibrillation (Af) and AMEI (*n* = 35; Af group) as controls. Serial D-dimer levels were measured between days 7 and 28 after stroke (sub-acute phase) in 21 patients of the TS group and 24 patients of the Af group.

**Results:** D-dimer levels at onset (acute phase) were significantly higher in the TS group (8.45 ± 1.79 μg/mL, *n* = 38) compared with the Af group (1.14 ± 0.14 μg/mL, *n* = 35) (*p* < 0.0001). In patients for whom serial D-dimer measurements were made, D-dimer levels measured at the sub-acute phase decreased to 0.48 ± 0.12 μg/mL (*n* = 24) in the Af group, but remained elevated in the TS group during the sub-acute phase (11.20 ± 2.77 μg/mL, *n* = 21) (*p* < 0.0001). In all TS patients in whom serial D-dimer measurements were made, D-dimer levels in 17 patients who died within 500 days (13.31 ± 3.23 μg/mL) were significantly higher than those of the four surviving patients (2.23 ± 0.38 μg/mL) (cut-off D-dimer level = 3.0 μg/mL) during this period. Moreover, serial D-dimer levels of 10 patients who died within 90 days (17.78 ± 4.60 μg/mL) were significantly higher than those of the 11 patients who survived up to 90 days (5.21 ± 2.12 μg/mL) (*p* < 0.05).

**Conclusions:** Serial D-dimer levels may be a good biomarker for TS as well as a useful predictor of the prognosis of TS patients.

## Introduction

The development of acute multiple embolic infarctions (AMEI) caused by cancer-associated hypercoagulopathy is termed as Trousseau's syndrome (TS) (1). Diagnosis of TS is largely based on the presence of multifocal simultaneous high-intensity lesions on diffusion-weighted imaging (DWI) on magnetic resonance imaging (MRI) (2–5) and hypercoagulability indicated by elevated D-dimer levels in the acute phase (2, 3, 6–9). However, various cut-off values of D-dimer levels have been reported for the differential diagnosis of TS patients (2, 7, 8). We recently reported that a D-dimer level ≥2.0 μg/mL was a useful diagnostic marker of TS, compared with patients with atrial fibrillation (Af) alone as a control group based upon Af being the major cause of AMEI (10). In the previous study, hypercoagulability with resistance to anticoagulation therapy in patients with both stroke and active cancer was associated with their poor prognosis (11). Furthermore, we found that the effect of anticoagulation therapy reduced D-dimer levels during the sub-acute phase in patients with Af alone, whereas D-dimer levels increased during the sub-acute phase and remained elevated despite anticoagulation therapy in TS patients (10). Thus, serial measurements of D-dimer levels in TS patients may be useful for diagnosing TS patients as well as for evaluating the efficacy of anticoagulant therapy. Moreover, D-dimer levels might be a good indicator for predicting the prognosis of TS patients.

In the present study, we examined serial D-dimer levels in TS patients during the subacute phase compared with patients with Af alone to validate the above assumption. Our present findings strongly suggest that the serial measurement of D-dimer levels is a useful predictor of the prognosis of TS patients.

## Materials and methods

### Subjects and demographic data

#### Patient selection

**Figure 1:**
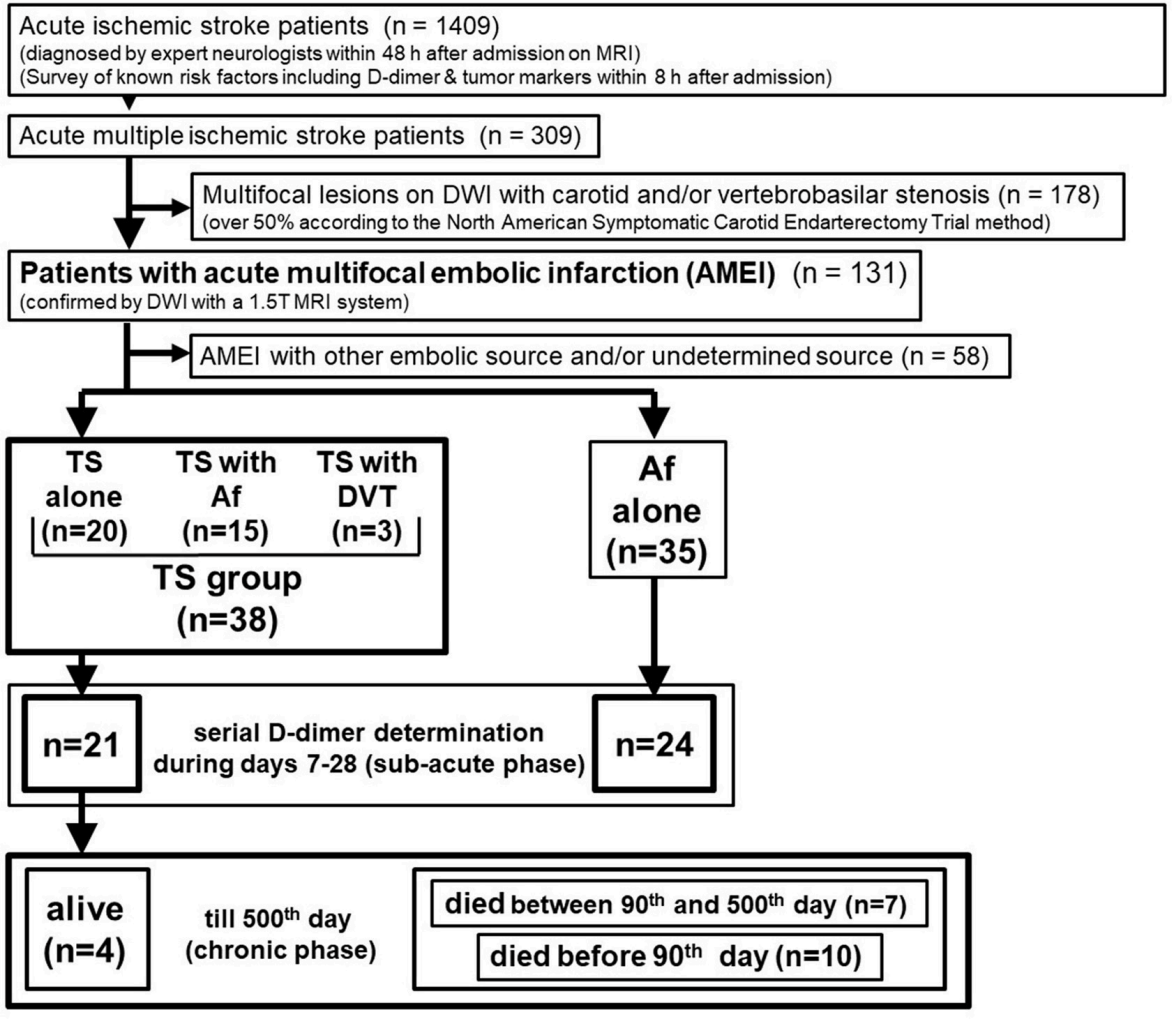
Patient selection. DWI, diffusion-weighted imaging of magnetic resonance imaging; Af, atrial fibrillation; TS, Trousseau's syndrome; DVT, deep vein thrombosis.

Precise inclusion and exclusion criteria are shown in Figure 1. Overall, 1,409 consecutive acute ischemic stroke patients were admitted to the stroke care unit in Fujita Health University Hospital between April 2011 and March 2016. Sequential MRI of the brain and head and neck MR angiography were performed for all patients, except for patients with pacemakers or magnetic materials in their body and those with contraindications such as claustrophobia or severe dementia that cannot remain in a restful state. Brain MRI analysis was performed by certified neurology and stroke experts and was double-checked by neuro-radiologists at our university hospital. Routine laboratory tests and coagulation studies, including serial D-dimer measurements, as well as a survey of known risk factors including tumor markers, were performed in all cases within 8 h after admission. We further examined patients who showed any increased tumor marker value by whole body computerized tomography (CT) scan for the search of occult malignancy. We identified 309 AMEI patients as those with multifocal infarcts detected by DWI on MRI within 48 h after admission, and 178 patients with carotid and/or vertebrobasilar arterial stenosis >50% were excluded, which is considered a likely major embolic source without a hypercoagulable state. Overall, 58 of 131 patients with AMEI caused by another embolic source and/or undetermined source were also excluded from this study. We identified 73 AMEI cases caused by TS [including cancer with Af or deep vein thrombosis (DVT), *n* = 38] or Af alone (i.e., control group, *n* = 35) who did not receive recombinant tissue plasminogen activator or endovascular therapy. These patients had been tracked up to 500 days after stroke onset, except in cases that died or who were transferred to other hospitals and clinics, which was mainly because of economic reasons, irrespective of the disease severity. Serial D-dimer levels during days 7–28 after stroke (sub-acute phase) were available for 24 patients with Af alone and 21 patients with TS. All patients provided written informed consent for the prospective use of clinical imaging and laboratory data for research by patients themselves or immediate relatives, and the study was approved by the ethical review board of Fujita Health University.

#### Clinical assessment

All patients were examined by DWI using a 1.5 T MRI system. MRI data were corrected on Signa^®;^ EXCITE™ /X1 1.5T system (GE Healthcare, Milwaukee, USA). Infarct sizes were measured automatically using medical viewing software (TFS-01 DV-R v1.95; Toshiba Medical Systems Corporation, Tochigi, Japan). Head and neck MR angiography and carotid ultrasonography (USG) were also performed. Overall, 131 patients who did not exhibit >50% carotid or vertebrobasilar arterial stenosis based on the North American Symptomatic Carotid Endarterectomy Trial criteria were included in the study. On the other hand, 58 patients with other embolic sources (except Af and cancer) were excluded, because they had various underlying diseases including cardiac valvular disease in ten patients, deep venous thrombosis with foramen ovale in six (without cancer and Af), myocardial infarction in five, cardiomyopathy in five, chronic heart failure caused by Af in three, non-bacterial thrombotic endocarditis without cancer in three, aortic dissection in three, infectious disseminated intravascular coagulation (DIC) in three, chronic heart failure with unknown etiology in two, left atrial thrombus in two, anti-neutrophil cytoplasmic antibody-associated vasculitis in two, systemic lupus erythematosus in two, and one case each of bacterial thrombotic endocarditis, atrial septal defect, hypercoagulopathy associated with hepatic cirrhosis, antiphospholipid antibody syndrome, rheumatoid arthritis, chronic thyroiditis, polycythemia vera, and amyloid angiopathy. Four cases were from an unknown associated disease.

In 73 patients with Af or TS, serial D-dimer values were obtained for 45 (62%) patients during the sub-acute phase. Serial D-dimer measurements in the sub-acute phase were performed 1–5 times according to the general condition of the patients, and the stable state values were used after treatment with heparin or oral anticoagulant. We adopted the latest value during the sub-acute phase to exclude the values affected by acute infections (e.g., aspiration pneumonia in seven, and urine tract infection in five, and fever of undetermined origin in three) as the serial D-dimer value during the sub-acute phase. D-dimer levels were measured using the latex agglutination method with a STACIA® automatic coagulation analyzer (Mitsubishi Chemical Medience, Tokyo, Japan).

We also performed cardiac USG, identified a history of previous anticoagulant therapy and underlying diseases, and determined the localization and maximum size of the infarction on DWI performed during the acute-phase. Af was diagnosed by continuous telemetric monitoring of electrocardiogram (ECG) and Holter ECG. ECG monitoring was performed for as long as possible (at least 72 h) to detect paroxysmal Af. All stroke patients were consecutively examined for cancer if any increased tumor marker values was detected in patients other than AMEI patients, although we did not encounter such patients in the present study. Cancer patients were diagnosed using tumor marker measurements (carcinoembryonic antigen, carbohydrate antigen (CA) 19-9, CA15-3, CA125, pro-gastrin-releasing peptide, squamous cell carcinoma related antigen, cytokeratin fragment 19, and prostate-specific antigen), whole body gallium scintigraphy, abdominal, and pelvic USG, as well as whole body CT. Final cancer diagnoses were confirmed in all cases following histopathologic biopsy or resected sample examination. Patients with complex cardiac thromboembolic sources (left atrial or ventricular thrombus, aortic or mitral stenosis, aortic or mitral valve replacement, and bacterial or non-bacterial thromboendocarditis) were diagnosed and excluded from the study based on a combination of ECG, myocardial damage biomarker level (CK-MB, myoglobin, troponin I), transthoracic cardiac USG, and brain natriuretic peptide elevation. Some patients with suspicious findings on transthoracic cardiac USG received additional transesophageal cardiac USG examination. DVT was detected using USG and CT. Three patients with DVT related to cancer were included in the TS group. Other associated diseases were identified using serum biochemical and immunologic markers and enhanced whole-body CT.

### Statistical analysis

Statistical analysis was performed using statistical software (JMP12; SAS Institute Inc., Cary, NC, USA). D-dimer levels between the groups were compared using the Mann–Whitney *U*-test. Because this was an exploratory analysis of a convenience sample, no pre-specified power calculation was performed. To calculate the D-dimer cut-off value to differentiate cancer-associated infarcts from cardioembolism because of Af, a receiver operator characteristic (ROC) curve was generated (MedCalc statistical software for Windows v.17.5; MedCalc Software, Mariakerke, Belgium).

## Results

### Patient demographics and characteristics of stroke and cancer

The characteristics of the 73 patients with AMEI are shown in Table 1. In the TS group, 28 patients (74%) exhibited acute infarcts on multiple vascular territories, which was significantly higher than that for 17 patients (49%) in the Af group, as previously reported (10). The infarcts in both groups were distributed almost equally on both internal carotid and vertebrobasilar territories. Ten patients (26%) in the TS group and 18 patients (51%) in the Af group exhibited acute multiple infarcts on a single vascular territory, and the distribution of these infarcts did not show any preponderance on each vascular territory similar to the infarcts on multiple vascular territories. There were no other differences in demographics between the two groups, except for a previous history of anticoagulant therapy.

**Table 1:** Demographics of patients with acute multifocal embolic infarcts.

	**Af alone**	**TS group**	***p*-value**
	**(*n* = 35)**	**(*n* = 38)**	
Age, years (mean ± SE)	74.7 ± 1.53	75.2 ± 1.66	n.s.
Male sex, *n* (%)	24 (74%)	23 (61%)	n.s.
NIHSS	4.74 ± 0.92	3.76 ± 0.44	n.s.
Body mass index (mean ± SE)	21.3 ± 0.59	21.2 ± 0.67	n.s.
Risk factors			
Hypertension	17 (49%)	17 (45%)	n.s.
Diabetes	11 (31%)	9 (24%)	n.s.
Hyperlipidemia	8 (23%)	10 (26%)	n.s.
Smoking^*^	13 (42%)	12 (35%)	n.s.
Preventive therapy for infarction			
Anticoagulant therapy	8 (23%)	2 (5%)	<0.05
Antiplatelet therapy	2 (6%)	5 (14%)	n.s.
Anticoagulant therapy after stroke	31 (89%)	31 (82%)	n.s.
Maximum size of lesion < 15 mm	23 (66%)	20 (53%)	n.s.
Infarcts on multiple vascular territories	17 (49%)	28 (74%)	<0.05
D-dimer value (μg/mL)			
On admission	1.14 ± 0.14	8.45 ± 1.79	<0.0001
During days 7–28^†^	0.48 ± 0.12	11.20 ± 2.77	<0.0001

*Af, atrial fibrillation; others, other diseases; SE, standard error; n.s., not significant*.

* *Smoking histories of nine patients (five with Af, four with cancer) could not be determined*.

† *D-dimer values between days 7 and 28 after stroke onset in 28 patients (11 with Af, 17 with cancer) could not be determined*.

D-dimer levels on admission are summarized in Table 1. D-dimer levels during the acute phase were significantly higher in the TS group than in the Af group. Fifteen TS patients were complicated by Af, and three were complicated by DVT. However, D-dimer values of TS patients with Af or DVT were similar to those we previously reported in TS patients without Af (10).

Cancers detected in the TS group were present in the following organs: colon in nine patients, pancreas in seven, lung in six, bile duct in five, uterus, and stomach in three patients each, and breast, gallbladder, prostate, thyroid, and osteosarcoma in one patient each. Clinical cancer stages were within stage III or IV in 25 cases. These patients received consultation from their respective oncologists for further examination and possible treatments. Prognoses were poor such that 21 cases died within 500 days (in 17 cases, serial D-dimer measurements were performed during the sub-acute phase) after stroke onset, while 11 cases died within 90 days (in 10 cases, serial D-dimer measurements were performed during the sub-acute phase). Except for one patient who died for DIC because of pneumonia, the causes of death in 20 cases were all related to cancer progression. Regarding cancer treatments, 14 cases with advanced cancer received only palliative therapy. AMEI was the initial cancer symptom in 15 (39%) patients in the TS group.

### D-dimer cut-off values for differential diagnosis of TS from AMEI associated with AF at onset and during the subacute phase

**Figure 2:**
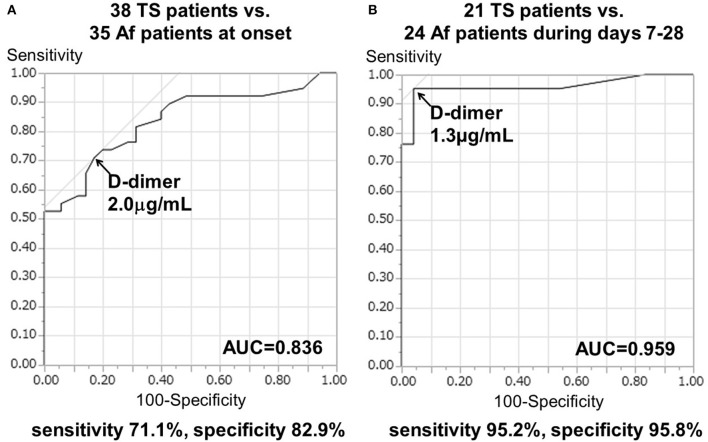
Receiver operating characteristic (ROC) curve analysis of D-dimer levels to differentiate the TS group from Af alone **(A)** at onset and **(B)** during days 7–28 after stroke onset. AUC, area under the ROC curve.

The cut-off D-dimer value, calculated by ROC curve analysis, was 2.0 μg/mL (odds ratio, 10.4; 95% confidence interval, 3.62–34.41; *p* < 0.0001), with a sensitivity of 71.1% and a specificity of 82.9% to differentiate TS (including 15 cases of TS with Af and three cases of TS with DVT) from cardioembolism caused by Af alone at onset. The area under the ROC curve (AUC) was 0.836 ± 0.048 (*p* < 0.0001; Figure 2A).

Serial D-dimer levels during days 7–28 after stroke (sub-acute phase) were available for 24 patients with Af and 21 patients with TS. Serial D-dimer values in the Af group decreased to 0.48 ± 0.12 μg/mL during the sub-acute phase, while those of the TS group remained elevated at 11.20 ± 2.77 μg/mL during the sub-acute phase. More importantly in the present study, the cut-off D-dimer value, calculated using ROC curve analysis, was found to be 1.3 μg/mL (odds ratio [OR], 382.2; 95% confidence interval [CI], 17.3–8434.4; *p* < 0.0005), with a sensitivity of 95.2% and a specificity of 95.8% to differentiate TS (including six cases of TS with Af and two cases of TS with DVT) from cardioembolism due to Af alone during the sub-acute phase. The AUC was 0.959 ± 0.034 (*p* < 0.0001; Figure 2B).

### Correlation of serial D-dimer values during the sub-acute phase with the prognoses of TS patients

**Figure 3:**
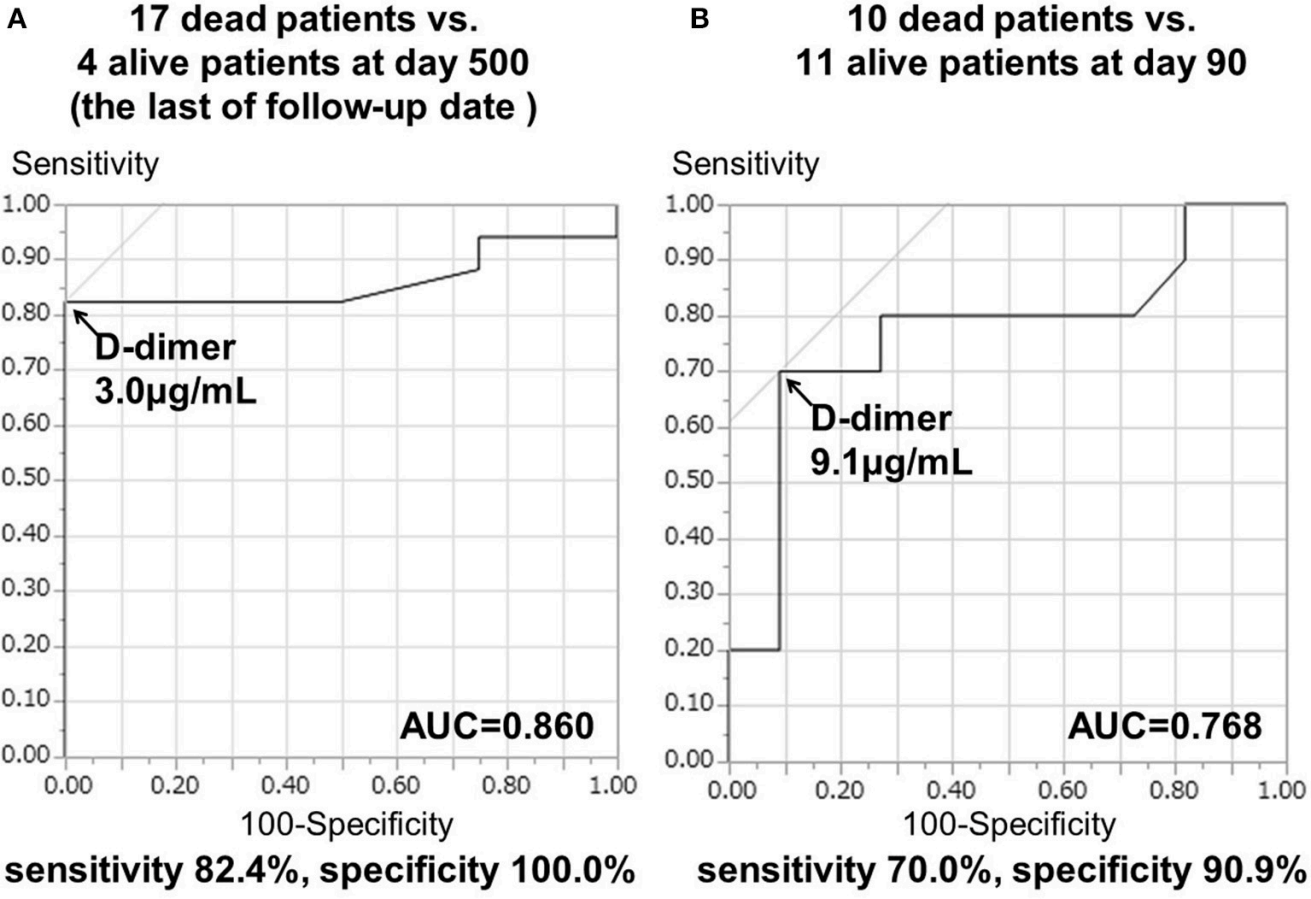
ROC curve analysis of D-dimer levels during days 7–28 and prognosis in TS patients. Repeated D-dimer measurements between days 7 and 28 were performed in 21 cancer patients. **(A)** AUC ± standard error (SE) to differentiate 17 deceased patients from four surviving patients at day 500. **(B)** AUC ± SE was used to differentiate 10 deceased patients from 11 surviving patients at day 90.

The 21 patients in TS group who had serial D-dimer measurements showed specific clinical characteristics (Supplementary Table 1). The main result of the present study was that the higher serial D-dimer values during the sub-acute phase correlated with the poor prognosis of TS patients as described below. The mean D-dimer levels during the sub-acute phase in 17 patients who died within 500 days (13.31 ± 3.23 μg/mL) were higher than those of four surviving TS patients (2.23 ± 0.38 μg/mL) (*p* < 0.05). Moreover, the mean D-dimer levels during the sub-acute phase of 10 patients who died within 90 days (17.78 ± 4.60 μg/mL) were significantly higher than those of the 11 surviving patients (5.21 ± 2.12 μg/mL) (*p* < 0.05). The cut-off D-dimer value during the sub-acute phase to differentiate deceased from surviving patients at the 500th day (chronic phase) was 3.0 μg/mL, with a sensitivity of 82.4% and a specificity of 100.0%. The AUC was 0.860 ± 0.082 (*p* < 0.0001; Figure 3A) and the 95% confidence interval was 0.640–0.971. Furthermore, the cut-off D-dimer value to predict death occurrence within 90 days was 9.1 μg/mL, with a sensitivity of 70.0% and a specificity of 90.9%. The AUC was 0.768 ± 0.116 (*p* < 0.0001; Figure 3B) and the 95% confidence interval was 0.535–0.922.

**Figure 4:**
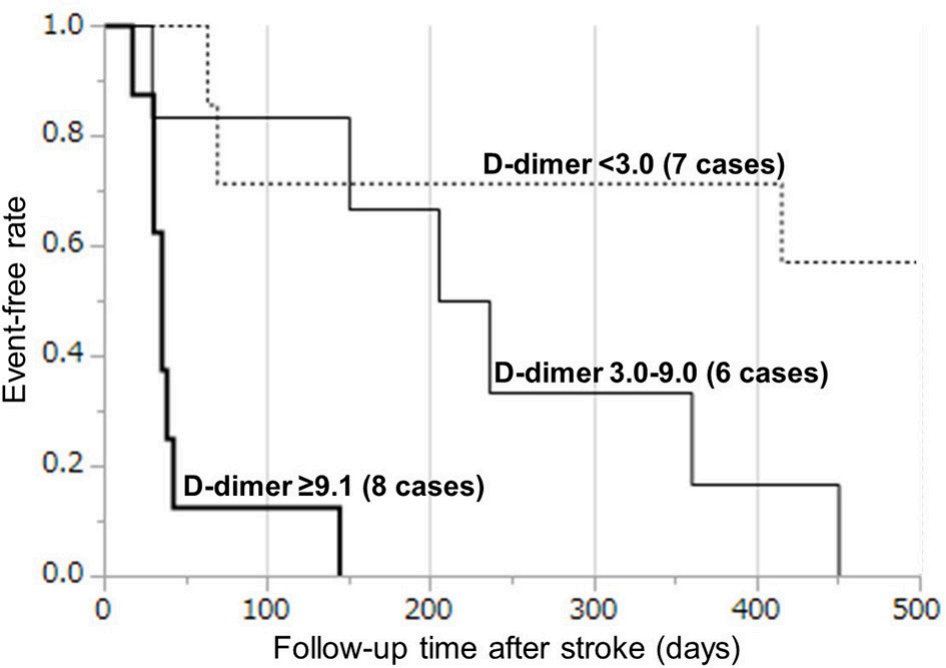
Kaplan–Meier curves by tertiles of D-dimer levels during days 7–28. The survival rate was analyzed by a Kaplan–Meyer survival curve using tertiles of D-dimer levels: (1) < 3.0 μg/mL, (2) 3.0–9.0 μg/mL, and (3) ≥9.1 μg/mL.

Based on the cut-off D-dimer values during the sub-acute phase, the survival rate was determined by a Kaplan–Meyer survival curve using tertiles of D-dimer levels (Figure 4). Kaplan–Meier curve analysis by the tertiles of D-dimer levels showed that patients in the 3rd tertile (D-dimer ≥9.1 μg/mL) had a higher risk than patients in the 1st tertile (D-dimer < 3.0 μg/mL) (log-rank test, *p* < 0.0001), while patients in the 2nd tertile (D-dimer 3.0–9.0 μg/mL) had a relatively higher risk than patients in the 1st tertile (Table 2). All patients in the 2nd and 3rd tertiles, except for one patient who died because of DIC, died within 500 days because of cancer progression (but not of stroke recurrence). Hazard ratio with cox-regression for mortality at the 500th day showed an increase in the 2nd and 3rd tertiles (3.78; *p* = 0.059 and *p* = 24.6; *p* < 0.0001, respectively) compared with the 1st tertile.

**Table 2:** Demographics of patients with cancer divided by tertiles of D-dimer values between days 7 and 28.

	**1st tertile DD < 3.0 (*n* = 7)**	**2nd tertile DD 3.0–9.0 (*n* = 6)**	**3rd tertile DD ≥ 9.1 (*n* = 8)**	***p*-value**
Age, years (mean ± SE)	78.6 ± 3.56	72.8 ± 3.91	75.1 ± 3.47	n.s.
Male sex, *n* (%)	3 (43%)	4 (57%)	6 (75%)	n.s.
NIHSS	3.14 ± 0.77	4.83 ± 7.74	4.13 ± 0.91	n.s.
Body mass index (mean ± SE)	17.7 ± 0.83	22.3 ± 1.84	20.4 ± 1.31	n.s.
Advanced stage of cancer	5 (71%)	6 (100%)	8 (100%)	n.s.
Died within 500 days	3 (43%)	6 (100%)	8 (100%)	<0.005
within 90 days	2 (29%)	1 (17%)	7 (88%)	<0.01
Risk factors				
Hypertension	3 (43%)	3 (50%)	3 (38%)	n.s.
Diabetes	0 (0%)	4 (67%)	1 (13%)	n.s.
Hyperlipidemia	1 (14%)	3 (50%)	2 (25%)	n.s.
Smoking	2 (29%)	1 (17%)	3 (38%)	n.s.
Preventive therapy for infarction				
Anticoagulant therapy	0 (0%)	0 (0%)	1 (13%)	n.s.
Antiplatelet therapy	0 (0%)	3 (50%)	1 (13%)	n.s.
Anticoagulant therapy after stroke	6 (86%)	4 (67%)	7 (88%)	n.s.
Maximum size of lesion < 15 mm	3 (43%)	2 (33%)	4 (40%)	n.s.
Infarcts on multiple vascular territories	5 (71%)	5 (83%)	6 (75%)	n.s.
D-dimer value (μg/mL)				
On admission	5.27 ± 1.84	6.87 ± 1.22	25.04 ± 4.91	<0.001
During days 7–28	1.77 ± 0.35	4.52 ± 0.65	24.45 ± 4.05	<0.0001
Recurrence of stroke	1 (14%)	2 (33%)	2 (25%)	n.s.

*SE, standard error; n.s., not significant*.

## Discussion

The main result of this study is that we found the clinical usefulness of D-dimer value during the subacute phase of the illness as a predictor of the prognosis in TS patients and confirmed the previous findings that D-dimer values are important diagnostic biomarker for TS in AMEI patients.

TS is a thrombotic syndrome caused by hypercoagulopathy associated with cancer (1), and there are several recent studies on TS as a cause of cryptogenic stroke, especially ESUS. For example, Navi et al. (12) reported that the survival rate of cancer patients with cryptogenic strokes was lower than that of patients who sustained strokes because of known causes within the TOAST classification (cardioembolism, large artery atherosclerosis, small vessel occlusion, and other determined causes). Furthermore, the authors showed that the cumulative rate of recurrent thromboembolism was greater in patients with adenocarcinomas compared with other histopathological cancer sub-types (13). Nezu et al. also reported that the recurrence rate of AMEI in multiple vascular territories was high, prognosis was poor, and that cancer was a relatively common cause of death in a 3-year follow up of 272 cases with cryptogenic stroke (14). These studies suggest that the prediction and prevention of embolic strokes in cancer patients may be important for increasing patient survival. In some cases, cerebral infarction is also the initial manifestation of cancer (15), as observed in the present study.

Although the embolic infarction associated with TS is an important mechanism causing treatment-resistance and poor prognosis, currently there are no definite diagnostic guidelines for TS. In the present study, we identified two common features in TS patients: 1) acute simultaneous multiple embolisms in multiple vascular territories, and 2) elevation of D-dimer levels. However, multiple embolisms are also common in cardiac embolism, while elevated D-dimer levels can be caused by venous thrombosis, antiphospholipid antibody syndrome, and some vasculitis, as we previously reported (10). Thus, more effective diagnostic and prognostic biomarkers are required.

In the present study, we demonstrated that the D-dimer values during the sub-acute phase of most patients with TS increased despite anticoagulation therapy. Furthermore, a cut-off D-dimer value >1.3 μg/mL could differentiate TS from Af patients during the sub-acute phase (with a higher AUC value on ROC analysis than at the acute phase). These data suggest that the serial measurements of D-dimer levels during the sub-acute phase may improve TS diagnosis. As serial D-dimer values during the sub-acute phase reflect both improvement in hypercoagulopathy and resistance to anticoagulation therapy, the prognostic prediction should be improved when using multiple values at different time points compared with a single time point value at the acute phase. Moreover, we also determined the cut-off values of serial D-dimer levels during the sub-acute phase, which are useful for predicting mortality in TS patients. In patients with subacute phase D-dimer levels ≥3.0 μg/mL, all cases eventually died. Of these patients, those with D-dimer levels >9.0 μg/mL had a higher probability of death within 90 days. Thus, patients with higher D-dimer levels should receive total management for the prevention of recurrent stroke, as well as intensive examination and treatment for cancer. In good agreement with our present results, Nam et al. also reported that in stroke patients with active cancers who died within 30 days, D-dimer values during days 2–9 (mean 4th day) after onset frequently increased compared with values at onset (16).

In the present study, AMEI was the initial cancer symptom in 15 (39%) out of 38 patients in the TS group. However, the primary organ of the cancer did not affect the prognoses in TS patients, although there may be a contribution related to respective cancer stage. Thus, the present study clearly indicates that serial D-dimer determinations are useful in identifying cancer-related stroke and predicting their prognoses in AMEI patients.

Our study had several limitations. First, the sample size was small. Therefore, we could not discuss about the changes in serial D-dimer value in AMEI patients caused by other underlying diseases and/or undetermined embolic sources, because the numbers of patients with each various disease except for TS and Af was too small to perform statistical analysis. Second, this was a retrospective study, and we could not measure D-dimer values during the sub-acute phase in some patients. Third, we only investigated patients who had multifocal lesions, while cancer-associated strokes can cause a single lesion. However, single infarction in cancer patients would be atypical, and it is difficult to distinguish cancer-related single infarction from conventional stroke based on the clinical information including MRI findings, unless the possibility of occult cancer was investigated. Therefore, occult cancer should be assessed in such patients. Our present study included checking tumor markers as listed earlier for all consecutively admitted patients with acute stroke and we have not detected such patients except for TS patients to date. Fourth, we could not establish the utility of anticoagulation therapy for patients with TS. Two guidelines on the efficacy of anticoagulation therapy against TS were published by the American Society of Clinical Oncology (17) and the National Comprehensive Cancer Network (18), respectively, both of which recommended subcutaneous low molecular weight heparin injection and warfarin administration for the prevention of DVT and pulmonary embolism. However, the efficacy of anticoagulation therapy for embolic stroke has not been established. Indeed, in our preliminary analysis, D-dimer values decreased once during the sub-acute phase in some TS patients receiving warfarin or heparin, while a few TS patients in whom D-dimer values did not increase even after the 28th day survived longer than 500 days, without reoccurrence of cerebral infarction. Further studies on a large cohort of TS patients as well as patients with single infarction are required to confirm the applicability of the present results.

## Ethics statement

This study was carried out with written informed consent from all subjects. All subjects gave written informed consent in accordance with the Declaration of Helsinki. The protocol was approved by the ethical review board of Fujita Health University.

## Author contributions

SI acquired and analyzed data and drafted the manuscript. AU, KK, RN, TM, KM, SS, and YM evaluated patients and participated in discussions. YN independently performed all statistical analyses. TM designed the study, provided scientific supervision, drafted, and revised the manuscript.

### Conflict of interest statement

The authors declare that the research was conducted in the absence of any commercial or financial relationships that could be construed as a potential conflict of interest.
