# Supplementary material for: Changes in Serial D-Dimer Levels Predict the Prognoses of Trousseau's Syndrome Patients
Source: Front Neurol. 2018 Jul 3;9:528. doi: 10.3389/fneur.2018.00528 (PMC6037767; doi:10.3389/fneur.2018.00528)
Supplement: Supplementary file 1 [file Table_1.PDF]

Supplementary Table. 1

Clinical characteristics of patients in the TS group with the serial D-dimer values during days 7-28

prognosis	diagnosis	age	sex	NIHSS	maximum size of infarct	D-dimer (μg/mL) On admission	D-dimer (μg/mL) during days 7-28	PT-INR on admission	primary cancer	thrapy for cancer	stage of cancer	meta-static lesion	recurrent stroke	risk factors (HT, DM, HL)	BMI	survival period after stroke onset	cause of death
Dead before 90th day	C alone	85	M	2	≥15mm	40.3	15.4	1.15	bile duct	Pa	IV	N	+	HT, DM, HL	24.7	30	C
	C alone	81	M	2	<15mm	39.3	44.9	1.10	lung	Pa	IV	N	+	HT, HL	19.1	30	C
	C alone	79	M	5	<15mm	38.6	35.2	1.09	stomach	Ch	IV	D	-	N	19.3	35	C
	C + Af	79	F	5	≥15mm	23.1	25.1	1.12	bile duct	Pa	IV	D	-	N	15.5	35	C
	C + Af	58	M	6	<15mm	22.8	23.8	1.32	bile duct	Ch	IV	D	-	N	19.5	42	C
	C alone	64	M	2	≥15mm	7.5	16.5	1.04	colon	Op	III	N	-	N	24.7	38	C
	C alone	80	F	2	<15mm	6.9	1.9	0.99	lung	Ch	IV	N	-	N	19.1	63	C
	C + Af	81	M	9	≥15mm	5.5	4.5	1.41	pancreas	Pa	IV	D	-	HT	21.6	29	C
	C + Af	84	F	9	≥15mm	4.7	9.1	0.96	pancreas	Pa	II	N	-	HT	16.2	17	DIC
	C alone	82	F	0	<15mm	4.3	1.4	1.06	stomach	Op	III	N	-	N	17.6	69	C
Dead during 91-500th day	C alone	71	M	2	<15mm	24.0	25.6	1.14	pancreas	Pa	III	N	-	N	24.2	144	C
	C + VT	57	M	0	≥15mm	9.3	5.1	1.02	pancreas	Ch	III	N	+	DM	17.7	360	C
	C + VT	81	F	4	≥15mm	8.6	3.1	0.96	uterus	Ch & Ra	IV	D	-	DM, HL	23.2	205	C
	C + Af	66	F	3	≥15mm	8.5	7.3	1.21	uterus	Pa	IV	D	+	HT, HL	27.1	236	C
	C + Af	78	M	2	<15mm	7.9	3.0	1.07	lung	Op,Ch,Ra	III	N	-	HT, DM, HL	27.5	450	C
	C alone	74	M	11	≥15mm	1.4	4.1	0.94	colon	Op	IV	D	-	DM	16.9	150	C
	C alone	83	M	5	<15mm	0.9	0.2	1.14	lung	Ch, Ra	III	N	-	HT	17.2	415	C
Alive	C alone	91	F	6	≥15mm	15.2	2.8	1.27	colon	Pa	II	N	-	HT	15.3	>500	AL
	C alone	69	M	4	≥15mm	5.6	2.9	1.04	prostate	Pa	I	N	-	N	16.7	>500	AL
	C alone	63	M	3	<15mm	2.1	1.9	0.90	colon	Op	III	D	-	HL	21.8	>500	AL
	C alone	82	F	2	≥15mm	1.9	1.3	0.91	colon	Op	III	N	+	HT	16.0	>500	AL

C, cancer; Af, atrial fibrillation; VT, venous thrombosis; M, male; F, female; NE, not evaluated;
Op, operation; Ch, chemothrapy; Ra, radiation thrapy; Pa, palliative therapy or observation; D, detected; N, not detected;
HT, hypertension; DM, diabetes mellitus; HL, hyperlipidemia; BMI, body mass index. DIC, disseminated intravascular coagulation ; AL, alive
